# Putative invasive pulmonary aspergillosis in critically ill patients with chronic obstructive pulmonary disease: a matched cohort study

**DOI:** 10.1186/s13054-015-1140-1

**Published:** 2015-12-03

**Authors:** Claire Delsuc, Aurélie Cottereau, Emilie Frealle, Anne-Lise Bienvenu, Rodrigue Dessein, Sophie Jarraud, Oana Dumitrescu, Marion Le Maréchal, Florent Wallet, Arnaud Friggeri, Laurent Argaud, Thomas Rimmelé, Saad Nseir, Florence Ader

**Affiliations:** Département d’Anesthésie et de Réanimation, Hôpital Edouard Herriot, Hospices Civils de Lyon, Lyon, France; Département de Réanimation médicale, Centre Hospitalier Régional Universitaire de Lille, Lille, France; Faculté de Médecine, Université de Lille, Lille, France; Laboratoire de Parasitologie et de Mycologie médicale, Centre Hospitalier Régional Universitaire de Lille, Lille, France; Laboratoire de Parasitologie et de Mycologie médicale, Centre de Biologie Nord, Hospices Civils de Lyon, Lyon, France; Laboratoire de Bacteriologie, Centre Hospitalier Régional Universitaire de Lille, Lille, France; Département de Microbiologie, Centre de Biologie Est, Hospices Civils de Lyon, Bron, F-69677 France; Inserm U1111, Centre International de Recherche en Infectiologie (CIRI), Université Claude Bernard Lyon 1, Lyon, France; Département de Microbiologie, Centre Hospitalier Lyon-Sud, Hospices Civils de Lyon, Pierre-Bénite, F-69495 France; Université de Lorraine, Université Paris Descartes, EA 4360 APEMAC, Nancy, France; Département d’Anesthésiologie et de Réanimation, Centre Hospitalier Lyon-Sud, Hospices Civils de Lyon, Lyon, France; Service de Réanimation médicale, Hôpital Edouard Herriot, Hospices Civils de Lyon, Lyon, France; Département des Maladies Infectieuses et Tropicales, Hospices Civils de Lyon, F-69004 Lyon, France; Département des Maladies Infectieuses et Tropicales, Hôpital de la Croix-Rousse, Hospices Civils de Lyon, 103 Grande-Rue de la Croix-Rousse, 69317 Lyon cedex 04, France

## Abstract

**Introduction:**

Patients with advanced chronic obstructive pulmonary disease (COPD) are at risk for developing invasive pulmonary aspergillosis. A clinical algorithm has been validated to discriminate colonization from putative invasive pulmonary aspergillosis (PIPA) in *Aspergillus*-positive respiratory tract cultures of critically ill patients. We focused on critically ill patients with COPD who met the criteria for PIPA.

**Methods:**

This matched cohort study included critically ill patients with COPD from two university hospital intensive care units (ICUs). We studied the risk factors for PIPA as well as the impact of PIPA on short- and long-term outcomes. Whether PIPA was associated with a pattern of bacterial colonization and/or infection 6 months before and/or during ICU stay was assessed. In addition, antifungal strategies were reviewed.

**Results:**

Fifty cases of PIPA in critically ill patients with COPD in the ICU were matched with one hundred control patients with COPD. The ICU short- and the long-term (at 1 year) mortality were significantly increased in the PIPA group (*p* < 0.001 for all variables). PIPA was a strong independent risk factor for mortality in the ICU (odds ratio 7.44, 95 % confidence interval 2.93–18.93, *p* < 0.001) before vasopressor therapy, renal replacement therapy, and duration of mechanical ventilation. Before ICU admission, the use of corticosteroids and antibiotics significantly increased the risk of PIPA (*p* = 0.004 and *p* < 0.001, respectively). No significant difference in bacterial etiologic agents responsible for colonization and/or infection was found between the groups. Antifungal treatment was started in 64 % of PIPA cases, with a poor impact on the overall outcome.

**Conclusions:**

PIPA was a strong death predictor in critically ill patients with COPD. The use of corticosteroids and antibiotics before ICU admission was a risk factor for PIPA. PIPA was not associated with a specific bacterial pattern of colonization or infection. Prompting antifungal treatment in critically ill patients with COPD who have PIPA may not be the only factor involved in prognosis reversal.

## Introduction

Invasive aspergillosis (IA) is a life-threatening opportunistic infection first described during prolonged neutropenia [1]. IA is associated with immunocompromised status that occurs in patients undergoing hematopoietic stem cell or solid organ transplants, in those with solid tumors, and in patients receiving corticosteroids [1–3]. In these settings, IA is defined by the European Organization for Research and Treatment of Cancer/Mycosis Study Group (EORTC/MSG) as proven, probable, or possible, based on a level of proof ranging from the decisive histopathological evidence of fungal invasion (proven) to a set of host risk factors and clinical features either associated (probable) or not (possible) with the positivity of mycological criteria [4].

Besides the immunocompromised setting, *Aspergillus* spp. can cause invasive disease in other subsets of patients, including those in intensive care units (ICUs) who may not fall into the above-described classifications [5, 6]. The mildly immunocompromised status of many non-malignant conditions, the nonspecific clinical and radiological patterns, and the limited value of mycological criteria led to reconsideration of the universal relevance of EORTC/MSG case definitions. Therefore, an alternative critically ill patient–adapted algorithm has been proposed to discriminate *Aspergillus* colonization from invasive pulmonary aspergillosis (IPA) [7, 8]. It has been externally validated by a multicenter study designed to confront the clinical criteria included in the algorithm with histopathology-proven cases of IPA [9]. Altogether, this algorithm has a specificity of 61 % and a sensitivity of 92 %. For an assumed IPA prevalence of 40 %, the positive and negative predictive values are 61 % and 92 %, respectively.

Chronic obstructive pulmonary disease (COPD) is one of the most common comorbidities among critically ill patients. Acute exacerbations cause frequent ICU admissions that may require mechanical ventilation (MV). In an observational multicenter study of 563 critically ill patients with proven putative invasive pulmonary aspergillosis (PIPA) or *Aspergillus* colonization, researchers found that COPD was the most frequent underlying condition, accounting for almost one-third (31 %, *n* = 174) of all cases [10]. Making an IPA diagnosis in ICU-admitted patients with COPD is challenging, as obtaining a lung biopsy is rarely feasible in patients with respiratory insufficiency on one side and *Aspergillus* spp. from endotracheal aspirate cultures may reflect colonization rather than infection on the other side.

We focused on critically ill patients with COPD who met the criteria for PIPA according to the clinical algorithm. A hypothesis was formulated that the algorithm may be a relevant tool for predicting the outcome of the specific subset of critically ill patients with COPD and PIPA. Using a matched cohort design, we investigated the short- and long-term outcomes of these patients. We sought to determine the risk factors for PIPA. In addition, we tested the hypothesis that PIPA may be associated with specific respiratory bacterial pathogens. Antifungal treatment strategies were also reviewed.

## Methods

### Study patients and case definitions

**Fig. 1:**
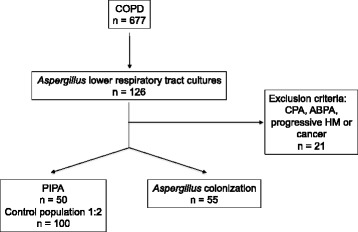
Flowchart of selected cases. *ABPA* allergic bronchopulmonary aspergillosis, *COPD* chronic obstructive pulmonary disease, *CPA* chronic pulmonary aspergillosis, *HM* hematological malignancy, *PIPA* putative invasive pulmonary aspergillosis

This retrospective study included critically ill patients admitted to the ICUs at the university hospitals of Lille and Lyon, France. Adult patients diagnosed with COPD according to the Global Initiative for Chronic Obstructive Lung Disease (GOLD) criteria between January 2006 and December 2013 and associated with a positive culture for *Aspergillus* spp. from a lower respiratory tract (LRT) specimen were identified in the computerized patient files at the two institutions (Fig. 1). Patients’ data were collected from a prospectively maintained database and by additional chart review. Patients eligible for inclusion were those who strictly matched the criteria for the PIPA algorithm [9]. PIPA referred to the presence of a positive culture for *Aspergillus* spp*.* from any LRT sample, together with meeting three criteria: (1) compatible signs and symptoms (fever refractory to at least 3 days of antibiotics, recrudescent fever, pleuritic chest pain or rub, dyspnea, hemoptysis, or worsening respiratory insufficiency despite adequate treatment), (2) abnormal medical imaging (chest x-ray or computed tomographic scan of the lungs), and (3) either host factor (neutropenia, underlying hematological or oncological malignancy treated with cytotoxic agents, glucocorticoid treatment, congenital or acquired immunodeficiency) or semiquantitative *Aspergillus*-positive culture of bronchoalveolar lavage (BAL) fluid, without concurrent bacterial growth, together with a positive cytological smear showing branching hyphae. Patients without any clinical or imaging findings or without host factor or cytological findings with positive cultures for *Aspergillus* spp*.* from an LRT sample were considered colonized. Patients with conditions such as chronic pulmonary aspergillosis, allergic bronchopulmonary aspergillosis, progressive hematological malignancy, or solid tumor were excluded (Fig. 1). Serum detection of galactomannan was not used as a diagnostic criterion in this study.

The control population in this 1:2 matched cohort study consisted of adult ICU patients with confirmed COPD and absence of *Aspergillus*-positive culture in respiratory samples. Cases were matched on age (±5 years), Simplified Acute Physiology Score (SAPS II) (±5 points), and ICU length of stay (equal to or more than the case until the diagnosis of PIPA). The study was approved by the local ethics committee of each participating center (Comité d’Ethique, Centre Hospitalier Régional Universitaire de Lille et Hospices Civils de Lyon). Because of the retrospective, observational nature of the study and the lack of any modification in the general management of these patients, the need for informed consent was waived.

### Data analysis

The primary objective of this study was to determine the prognostic factors for PIPA. ICU length of stay, duration of MV, short-term mortality (in the ICU and at 28 days after ICU admission), and long-term mortality (at 1 year after ICU admission) were used as endpoints.

The secondary objective of the study was to determine the risk factors for PIPA in critically ill patients with COPD admitted to the ICU. We included the following variables: demographic variables, underlying diseases, COPD characteristics, SAPS II and Logistic Organ Dysfunction System scores upon admission and on the day of the *Aspergillus* spp.–positive culture, corticosteroid antibiotic use, and supportive therapies. Radiological and clinical data were collected according to PIPA criteria [9].

The third objective of the study was to assess whether PIPA was associated with a pattern of bacterial colonization or infection 6 months before and during the ICU stay. The presence of culture-positive sputum or LRT specimens with the following specific bacterial pathogens was investigated: respiratory Gram-negative bacteria (*Haemophilus influenzae* and *Moraxella catarrhalis*), enterobacteria, non-fermenting Gram-negative bacilli (*Pseudomonas* spp., *Acinetobacter* spp., *Stenotrophomonas* spp., *Burkholderia* spp.), and Gram-positive cocci (*Staphylococcus aureus*, *Streptococcus pneumoniae*, *Enterococcus* spp*.*). In addition, antifungal strategies applied to critically ill patients with COPD in the ICU with PIPA were examined through the delay of initiation and the drug(s) chosen for treatment.

### Statistical analysis

IBM SPSS software (IBM, Armonk, NY, USA) was used for data analysis. Categorical variables were described as frequencies and percentages. The distribution of continuous variables was tested for normality. Normally distributed and skewed continuous variables were described as mean ± standard deviation or as median and interquartile range (IQR), respectively. All *p* values were two-tailed. Differences were considered significant if *p* values were <0.05.

To determine factors associated with PIPA, patients with PIPA were compared with those without PIPA using bivariate and multivariate conditional logistic regression analyses. All variables from univariate analysis with *p* values <0.1 were incorporated into the multivariate logistic regression analysis. Potential interactions were tested, and the Hosmer-Lemeshow goodness of fit was calculated. The odds ratio (OR) and 95 % confidence interval (CI) were calculated for all significant variables in univariate analysis as well as all significant variables in multivariate analysis.

To determine the impact of PIPA on mortality, survivors and non-survivors among patients with PIPA were compared using univariate and multivariate analyses. The χ^2^ test or Fischer’s exact test was used to compare qualitative variables, as appropriate. Student’s *t* test or the Mann–Whitney *U* test was used to compare normally distributed and skewed continuous variables, as appropriate. All variables from univariate analysis with *p* values <0.1 were incorporated into the multivariate logistic regression analysis. Potential interactions were tested, and the Hosmer-Lemeshow goodness of fit was calculated. The OR and 95 % CI were calculated for all significant qualitative variables in univariate analysis and for all significant variables in multivariate analysis. Further on, the probability of death in the ICU over time was assessed using the Kaplan-Meier estimate and the log-rank test.

Univariate analysis between case and control patients was performed to compare the presence of culture-positive sputum or LRT specimens with specific bacterial pathogens. We used a χ^2^ test or Fisher’s exact test, as appropriate. The OR and 95 % CI were calculated for all variables. No multivariate regression was performed on these data.

## Results

### Patient characteristics and clinical presentation

Fifty cases of PIPA in critically ill-patients with COPD were identified between 2006 and 2013. These patients comprised 38 men and 12 women with a median age of 67 (IQR 60–74) years at diagnosis. The patients’ baseline characteristics are summarized in Table 1. As all cases had positive *Aspergillus*-LRT cultures, *A. fumigatus* was identified as the pathogen in 46 cases (92 %) and other *Aspergillus* spp. in 4 cases (8 %). Microscopy of tracheal aspirates or BAL fluid demonstrated fungal hyphae in 16 (32 %) of 50 samples.

**Table 1:** Patient characteristics according to putative invasive pulmonary aspergillosis diagnosis

	All patients (*n* = 150)		PIPA (*n* = 50)		No PIPA (*n* = 100)		*p* Value^a^
	Number (%)	NA (*n*)	Number (%)	NA (*n*)	Number (%)	NA (*n*)	
Demographics							
Male sex, *n* (%)	111 (74)		38 (76)		73 (73)		0.52
Age, yr (IQR)	66 (60–73)		67 (60–74)		66 (70–73)		0.69
Weight, kg (IQR)	77 (65–94)	19	72 (62–87)	7	78 (67–100)	12	0.098
Underlying conditions, *n* (%)							
Diabetes mellitus	34 (22.7)		8 (16)		26 (26)		0.21
Chronic heart failure	25 (16.7)		11 (22)		14 (14)		0.61
Chronic renal failure	6 (4)		2 (4)		4 (4)		1
Chronic liver failure	8 (5.3)		4 (8)		4 (4)		0.31
Solid tumor (CR)	35 (23.3)		11 (22)		24 (24)		0.74
Hematological malignancy (CR)	5 (3.3)		4 (8)		1 (1)		0.57
Autoimmune disease	6 (4)		3 (6)		3 (3)		0.39
Alcohol abuse	42 (28)		14 (28)		28 (28)		1
COPD characteristics							
GOLD grade ≥3, *n* (%	78 (62.9)	26	31 (68.9)	5	47 (59.5)	21	0.30
FEV_1_ % (IQR)	41 (29–59)	30	37 (27–57)	8	43 (30–60)	22	0.47
Smoking history, *n* (%)	135 (93.7)	6	43 (93.5)	4	92 (93.9)	2	0.92
Pack-years (IQR)	40 (30–60)	61	40 (30–58)	19	45 (30–60)	42	0.66
Acute exacerbations,^b^ *n* (%)	28 (21.7)	21	8 (17.8)	5	20 (23.8)	16	0.54
Corticosteroid use, *n* (%)							
Chronic use >3 mo	22 (14.7)		13 (26)		9 (9)		0.004
Daily dose >20 mg	77 (52.7)		36 (73.5)		41 (42.3)		<0.001
Chronic inhalational corticosteroid use	75 (51)	3	28 (56)		47 (48.4)	3	0.52
ICU admission							
Antibiotics (prior 3 months)	82 (55.4)	2	40 (83.3)	2	42 (42)		<0.001
SAPS II, mean (SD)	46.9 (13.9)		46.9 (13.9)		47 (13.9)		0.99
LODS score (IQR)	6 (4–8)		6 (3–7)		6 (4–8)		0.26
Supportive therapy in ICU							
MV duration	14 (5–27)		18 (8–26)		12 (5–26)		0.60
Renal replacement therapy, *n* (%)	25 (17)		7 (14)		18 (18)		0.57
Vasopressive or inotropic agents, *n* (%)	95 (63)		32 (64)		63 (63)		0.69
Symptoms, *n* (%)							
Refractory fever	17 (11.3)		6 (12)		11 (11)		0.85
Recrudescence of fever	19 (12.7)		11 (22)		8 (8)		0.02
Pleural effusion	17 (11.3)		6 (12)		11 (11)		0.85
Dyspnea	136 (90.7)		49 (98)		87 (87)		0.07
Hemoptysis	8 (5.3)		5 (10)		3 (3)		0.187
Worsening of respiratory insufficiency	50 (33.3)		30 (60)		20 (20)		<0.001

*PIPA* putative invasive pulmonary aspergillosis, *NA* not available, *IQR* interquartile range, *CR* complete remission, *COPD* chronic obstructive pulmonary disease, *GOLD* Global Initiative for Chronic Obstructive Lung Disease, *FEV*_*1*_ forced expiratory volume in 1 second, *MV* mechanical ventilation, *ICU* intensive care unit, *SAPS II* Simplified Acute Physiology Score, *LODS* Logistic Organ Dysfunction System, *SD* standard deviation

^a^ *p* < 0.05 was considered statistically significant

^b^At least three acute exacerbations or a single one requiring ICU hospitalization over the past year

### Survival, clinical features, and supportive therapies associated with PIPA

**Fig. 2:**
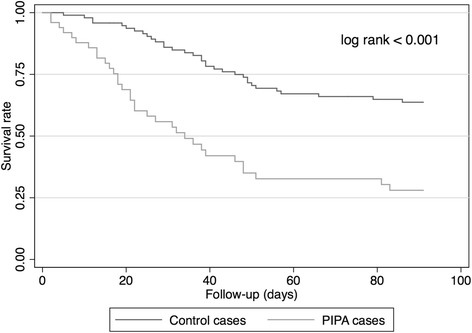
Kaplan-Meier curves showing survival rate in patients with putative invasive pulmonary aspergillosis (PIPA) and control patients over a 3-month time course after intensive care unit admission. *p* < 0.001 by log-rank test

In univariate analysis, mortality was significantly increased in the PIPA group in comparison with the control group when we considered either overall ICU mortality or short-term 28-day mortality (62 % vs. 29 % and 48 % vs. 16 %, respectively; *p* < 0.001 for both variables). The long-term mortality at 1 year after ICU admission was significantly increased in the PIPA group in comparison with the control group as well (74 % vs*.* 36 %, *p* < 0.001). The Kaplan-Meier statistics showed a significantly increased probability of death over time (*p* < 0.001 by log-rank test) (Fig. 2). The multivariate analysis confirmed PIPA as being a strong independent risk factor for mortality in the ICU (OR 7.4, 95 % CI 2.9–18.9, *p* < 0.001), as were vasopressor therapy (OR 5.6, 95 % CI 2.1–15.1, *p* = 0.001), renal replacement therapy (OR 5.3, 95 % CI 1.6–17.3, *p* = 0.005), and longer duration of MV (OR 1 per day of MV, 95 % CI 1–1.1, *p* = 0.004, Hosmer-Lemeshow goodness-of-fit test *p* = 0.798) (Table 2).

**Table 2:** Risk factors for mortality in ICU in multivariable analysis

Risk factor	All patients (*n* = 150)		
	OR	CI	*p* Value^a^
PIPA	7.4	2.9–18.9	<0.001
Vasopressor therapy	5.6	2.1–15.1	0.001
RRT	5.3	1.6–17.3	0.005
Duration of mechanical ventilation	1	1–1.1	0.004

*PIPA* putative invasive pulmonary aspergillosis, *OR* odds ratio, *CI* confidence interval, *RRT* renal replacement therapy

^a^ *p* < 0.05 was considered statistically significant; Hosmer-Lemeshow goodness-of-fit test *p* = 0.798

### Risk factors associated with PIPA

In univariate analysis, the underlying conditions of patients or their COPD characteristics were not risk factors for PIPA (Table 1). In multivariate analysis, the use of corticosteroids for longer than 3 months as well as the use of antibiotics before ICU admission significantly increased the risk of PIPA (OR 8, 95 % CI 2–31.9, *p* < 0.001; and OR 13.4, 95 % CI 4.3–41.8, *p* < 0.001, respectively; Hosmer-Lemeshow goodness of fit *p* = 0.824). The same significant result was obtained when we tested a prednisone equivalent dose >20 mg/day (data not shown).

### Bacteria-associated colonization or infection

To investigate whether the occurrence of PIPA was associated with specific bacterial species, we compared the colonization or infections occurring in patients with PIPA and control patients as long as 6 months before ICU admission (Table 3). Particular attention was paid to non-fermenting bacterium *Pseudomonas aeruginosa*, the presence of which among other Gram-negative bacteria was specifically documented. No significant difference in bacterial colonization or infection was found between the two groups.

**Table 3:** Bacteria-associated colonization or infection before or during PIPA

	All patients	PIPA	No PIPA	*p* Value^a^
	(*n* = 150)	(*n* = 50)	(*n* = 100)	
Positive airways culture, *n* (%)	91 (61)	30 (60)	61 (61)	0.725
Gram-negative bacteria, *n* (%)				
Respiratory^b^	14 (9)	1 (2)	13 (13)	0.054
Enterobacteria	35 (23)	9 (18)	26 (26)	0.236
Non-fermenting bacteria^c^	51 (34)	20 (40)	31 (31)	0.331
*Pseudomonas* spp.	38 (25)	14 (28)	24 (24)	0.670
Gram-positive bacteria, *n* (%)				
*Staphylococcus aureus*	21 (14)	10 (20)	11 (11)	0.160
Other cocci^d^	21 (14)	5 (10)	16 (16)	0.290

*PIPA* putative invasive pulmonary aspergillosis

^a^ *p* < 0.05 was considered statistically significant

^b^ *Haemophilus influenzae* and *Moraxella catarrhalis*

^c^ *Pseudomonas* spp., *Acinetobacter* spp., *Stenotrophomonas* spp., *Burkholderia* spp.

^d^ *Streptococcus pneumoniae*, *Enterococcus* spp.

### Antifungal regimen for PIPA

Overall, 32 patients (64 %) received an antifungal therapy, all of which were single-drug regimens. The antimold drugs used were voriconazole (59.4 %, *n* = 19), caspofungin, (28.1 %, *n* = 9), and amphotericin B (3.1 %, *n* = 1). Of note, 3 patients (9.4 %) received off-mold spectrum fluconazole. The median delay of antifungal treatment initiation was 8 (IQR, 5–11) days after ICU admission and 4 (IQR, 0–8) days after *Aspergillus* spp.–positive LRT specimen. Regarding the outcome of the treated patients, 20 (62.5 %) died in the ICU and the 1-year overall mortality rate was 75 % (*n* = 24). In the untreated group (*n* = 18), 11 (64.7 %) died in the ICU and the 1-year overall mortality rate was 76.5 % (*n* = 13).

## Discussion

We have demonstrated in critically ill patients with COPD a very significant association between PIPA and fatal outcome with follow-up of 12 months. PIPA was a stronger independent death predictor than other major specific ICU interventions (vasopressor use, renal replacement therapy, or duration of MV during ICU stay). In the setting of COPD, the present study confirms the usefulness of the PIPA algorithm to predict lethal outcome. A recent study of 297 critically ill patients with proven IPA or PIPA provided evidence in multivariate analysis that both MV and renal replacement therapy at diagnosis were risk factors for mortality [10]. In the present study, the 1-year mortality rate of 74 % is consistent with two previous COPD-focused case–control studies, although overall death was evaluated earlier in both studies (71.6 % at 4 months and 61.5 % at 6 months) [11, 12] than in our study. The use of an antifungal treatment did not make any difference in terms of short- and long-term mortality with a relatively short time to treatment initiation after ICU admission, indicating that prognosis is not exclusively linked to a matter of promptly implementing the appropriate anti-infectious treatment against *Aspergillus* spp. infection but may be multifactorial, related to overall severity of the patient’s condition. This point is in accordance with the results of previous studies showing that GOLD grades 3 and 4 are likely represented in the probable IPA group rather than in the *Aspergillus* spp.–colonized group [12, 13].

The use of antibiotics and high cumulative doses of corticosteroids significantly increased the risk of PIPA in critically ill patients with COPD with *Aspergillus* LRT cultures, as previously reported [10–16]. An originality of the present study is that we investigated whether PIPA occurrence is associated with specific bacterial communities, since very few data are available on cross-kingdom interactions. Particularly, we focused on *P. aeruginosa*, which is frequently found in advanced COPD and is known for its great mutational and biofilm-producing abilities, conferring increased antibiotic resistance [17]. An *in vitro* study has demonstrated a competitive inhibition of *A. fumigatus* biofilm formation by a set of *P. aeruginosa* strains [18]. However, the bacterial vacancy resulting from antibiotics may lead to further fungal biofilm-organized invasion and decreased susceptibility to antifungal drugs, as *Aspergillus* spp. has the ability to form a biofilm [19–21].

Strengths of the present study include a matched cohort design and the long-term follow-up of patients with PIPA. Limitations of the present study include the retrospective, nonconsecutive series and the bias resulting from the low number of enrolling centers as well as the low number of patients enrolled, PIPA being a rare entity (7.4 % of the critically ill patients with COPD were screened for the study over an 8-year period, which represents three cases per ICU per year). As the case fatality rate is high for ICU patients with COPD with PIPA, the lack of systematic postmortem autopsy or lung biopsy for histological confirmation mitigates the evidence-based value of the algorithm. In addition, mortality might have been overestimated because of the relatively small number of patients with PIPA. Stratification of mortality results according to GOLD grades could not be performed, owing to too many missing data for functional status at admission. Finally, the small number of treated patients hampers the conclusions regarding the impact of antifungal treatment on the outcome. Another bias related to the treatment is the lack of serum concentrations of antifungal drugs administrated to patients with PIPA, namely voriconazole, the data for which were not available.

## Conclusions

In the setting of critically ill patients with COPD, using a clinical algorithm designed to discriminate *Aspergillus* spp. colonization from PIPA, we could determine that PIPA was a strong predictor of lethal outcome in patients with COPD. Our data suggest that implementing an antifungal treatment may not be the only factor involved in prognosis reversal. PIPA was not associated with a dominant pattern of bacterial colonization or infection. In the setting of critically ill patients with COPD and PIPA, further prospective studies are required to assess relevant diagnostic methods, therapeutic support, and/or interventions in improving outcomes.

## Key messages

- PIPA, as defined according to the Blot et al. criteria [9], is a strong predictor of short- and long-term lethal outcome in critically ill patients with COPD.
- The use of antibiotics and high cumulative doses of corticosteroids significantly increased the risk of PIPA occurrence in critically ill patients with COPD with *Aspergillus* lower respiratory tract cultures.
- Prompting antifungal treatment in critically ill patients with COPD with PIPA may not be the only factor involved in prognosis reversal.
- PIPA was not associated with a dominant pattern of bacterial colonization or infection 6 months before or during fungal infection.

## Abbreviations

BAL: bronchoalveolar lavage
CI: confidence interval
COPD: chronic obstructive pulmonary disease
CR: complete remission
EORTC/MSG: European Organization for Research and Treatment of Cancer/Mycosis Study Group
FEV_1_: forced expiratory volume in 1 second
GOLD: Global Initiative for Chronic Obstructive Lung Disease
IA: invasive aspergillosis
ICU: intensive care unit
IPA: invasive pulmonary aspergillosis
IQR: interquartile range
LODS: Logistic Organ Dysfunction System
LRT: lower respiratory tract
MV: mechanical ventilation
OR: odds ratio
PIPA: putative invasive pulmonary aspergillosis
RRT: renal replacement therapy
SAPS II: Simplified Acute Physiology Score
SD: standard deviation
